# Mental health in Syrian children with a focus on post-traumatic stress: a cross-sectional study from Syrian schools

**DOI:** 10.1007/s00127-018-1573-3

**Published:** 2018-08-06

**Authors:** Jon Davis Perkins, Maiss Ajeeb, Lina Fadel, Ghassan Saleh

**Affiliations:** 1 PMARC, University of Edinburgh, St Leonard’s Land, Holyrood Road, Edinburgh, EH8 8AQ UK; 2 Department of Counselling, Damascus University, Damascus, Syria; 3 School of Social Sciences, Heriot-Watt University, Edinburgh, UK

**Keywords:** War trauma, PTSD, Depression, Anxiety, Risk factors

## Abstract

**Purpose:**

Studies show that conflict can negatively affect psychological health. The Syrian crisis is 8 years old and yet little is known about the impact of the conflict on the well-being of Syrians who remain. This gap was addressed by conducting an empirical study on the mental health burden of Syrian children in two areas of the country.

**Methods:**

492 children between 8 and 15 years were randomly selected from schools in Damascus and Latakia. The incidence of psychological disorder symptoms was measured using self-report screening instruments, the Children’s Revised Impact of Event Scale (CRIES-8) and the Revised Children’s Anxiety and Depression Scale (RCADS-25). Simultaneously, sociodemographic and traumatic event information was collected. Binary logistic regression was used to identify factors that influence the development of post-traumatic stress disorder (PTSD) symptoms.

**Results:**

In our sample, 50.2% of students were internally displaced and 32.1% reported a negative experience. 60.5% of those tested had at least one probable psychological disorder with PTSD the most common (35.1%), followed by depression (32.0%), and anxiety (29.5%). Binary logistic regression indicated that PTSD symptoms were predicted by: living in Damascus [odds ratio (OR) 2.36, 95% confidence interval (CI) 1.51–3.69], being female (1.54, 1.02–2.34), having depression and anxiety (2.55, 1.48–4.40), and the negative experiences; displacement and daily warzone exposure (1.84, 1.02–3.30 and 2.67, 1.08–6.60).

**Conclusions:**

Syrian children are experiencing traumatic events and war-associated daily stresses that are hugely impacting psychological well-being. Our data offer guidance for mental health providers regarding risk factors and highlights the use of the school system to reach suffering children.

## Introduction

The psychological well-being of young people is a primary global public health concern [1]. During times of conflict, children are particularly vulnerable to mental health issues. The crisis in Syria has entered its eighth year with no sign of a political solution emerging soon. The crisis has had a devastating impact on the country’s population with a plethora of negative factors present that affect psychological health. Many have been killed and/or injured, millions have been displaced including inside the country [internally displaced persons (IDPs)], and countless people are in need of humanitarian assistance [2, 3]. At the same time, the Syrian healthcare system has contracted, increasing pressure on those providing Syria’s mental health and psychosocial support (MHPSS). The country’s education and financial sectors have been severely affected. In some areas, many children no longer attend school [4]. Poverty and disease have increased and basic goods, fuel and medicines are limited with shortages exacerbated by United States (US) and European Union sanctions [5, 6]. Perhaps most importantly for child development and well-being, family and social structures have been damaged and younger Syrians below the age of eight will have grown up knowing nothing but war [7].

It has long been established that intervention can reduce the psychological impact of traumatic events [8]. Given the extent of the problems facing Syrian children, epidemiological studies are desperately required as a critical first step in understanding the psychological needs of the country’s youth. However, a feature of the Syrian conflict has been difficulty obtaining reliable data from inside the country and to date, no systematic empirical studies of child mental health have been carried out despite the recognition of their importance [9, 10]. Instead, most of the information regarding Syrians comes from areas held by armed groups which represent a fraction of the country’s population. The vast majority of Syrians live in Government-controlled areas and form the biggest challenge for MHPSS providers.

Studies with refugees in proximal countries and Europe have been the best indicators so far of the child mental health situation inside Syria [11, 12]. Nevertheless, refugee mental health may differ significantly compared to those living in conflict zones. Strong predictors of psychopathology such as ‘perceived threat to life’ or repeated negative experiences are diminished. However, research has shown that non-traumatic war-related daily stressors such as the breakdown of support networks or increased poverty, can influence mental well-being in the same way as direct trauma [13]. Refugee and warzone populations will have shared, but also unique, daily stressors and further study is required to understand how these factors impact child psychopathology.

The aim of this research is to document the mental health of Syrian children living inside the country. Due to its high prevalence in war settings, particular interest is paid to post-traumatic stress disorder (PTSD) and the factors that influence susceptibility to its development. In line with current practice in mental health research, this means identifying both the traumatic and psychosocial predictors of psychopathology.

## Methods

### Screening instruments and study design

Post-traumatic stress symptoms were screened using the Arabic Children’s Revised Impact of Event Scale (CRIES-8) [14]. The instrument has eight questions referring to intrusion and arousal as specified by the Diagnostic and Statistical Manual of Mental Disorders-4th edition [15]. Participants rate statements about thoughts in the past week, related to a previously experienced event such as, “Do you think about it even when you don’t mean to?”. Answers are given using a 4-point Likert scale ranging from 0 (none) to 5 (a lot). Scores ≥ 17 (for intrusion and avoidance combined) indicate probable PTSD and the need for further assessment [14]. CRIES-8 has been translated into Arabic and used with children who have experienced conflict [16].

To assess mood disorder, a shortened version of the Revised Children’s Anxiety and Depression Scale was used (RCADS-25) [17]. RCADS-25 has ten items relating to depression and 15 for anxiety. A unidimensional factor (anxiety and depression together) captures a higher order construct representing a general negative effect. All scale items relate to DSM-IV classifications [15]. Intended for youths in grades 3–12, the RCADS-25 asks participants to rate statements such as “I worry that something awful will happen to someone in my family” using a 4-point Likert scale. Scores range from 0 (never) to 3 (always) and clinical significance is assessed using *T* scores after raw data have been adjusted for sex and year of schooling. The scale has been tested in clinical and school-based populations, translated into Arabic and shows good cross-cultural utility [17, 18].

Ethical approval was secured from the Faculty of Education at Damascus University where both the Psychology and Counselling Departments are situated. After agreement from the Education Directorates of Damascus and Latakia, 15 state-run primary and secondary schools were selected at random in different districts of each city. Schools were included on a first-come-first-served basis after agreement from head teachers, with five schools in Damascus and three in Latakia participating. Written informed consent was obtained from parents or guardians and children were asked to consent verbally after the instruments were explained. Children were informed of their right not to answer questions they found uncomfortable and to withdraw from the research at any time. Responses were anonymized and the study was entirely voluntary with no incentives given. Screening instruments were compiled into a single questionnaire and given to students via paper during school hours with a teacher and an Arabic speaking member of the research team present. Information was provided regarding local and Web-based mental health providers in case taking part in the study raised concerns among participants. Psychologists/counsellors on the team also made themselves available for inquiries post-testing. Completion of the questionnaire took approximately 20 min with sociodemographic information collected simultaneously.

### Participants

Participants were selected from grades 3–9 with the lower age category specified by the screening instruments. According to Syrian Ministry of Education statistics, the combined Latakia and Damascus population of grade 3–9 students in 2016 was 356,741. To determine sample size it was reasoned that sub-populations of interest may have endured similar experiences during the conflict and that therefore, a sample large enough to detect small effect sizes was preferable (*d* = 0.3, *α* = 0.05, *β* = 0.85, two-tailed). This reasoning reflected the paucity of mental health studies conducted in actual warzones. A total of 384 was estimated and to account for dropout, an overall number approximately 10% above this figure was pursued. A minimum of 15 girls and 15 boys in each grade (maximum 40) was taken in both cities for a total cohort of 420 students (210 students per site). All children in participating schools were eligible to take part.

Data were collected in Damascus (the capital) and Latakia. Areas of the south, east, and far north of the country were excluded for access and security reasons. Cities were selected based on economic, cultural and historic diversity. Both sites share similar recent histories with big population increases from IDPs and large-scale terrorist attacks, including car and suicide bombings. However, in contrast to Latakia, at the time of writing frontlines existed within Damascus’s city limits. Parts of the east and south of the capital were under the control of armed groups, including Islamic State, for several years with frequent rocket and mortar fire reaching the city centre from these areas. In addition, the sounds of war such as aircraft, tank, artillery and gunfire, were clearly audible around the city and parts of the suburbs have been destroyed.

### Statistical analysis

IBM SPSS Statistics for Windows, version 22 was used for all data analysis [19]. To better understand the characteristics of our sample, distributions of sub-populations (e.g. internally displaced, social status, etc.) were explored by sex and data collection sites using Chi-square crosstabs. Bonferroni corrections were applied to account for multiple comparisons. Similarly, to recognize how different subgroups scored on screening instruments, independent *t* tests were employed. To identify risk factors for PTSD symptoms, univariable associations with a binary CRIES-8 outcome of no PTSD/probable PTSD were explored using binary logistic regression. Based on the existing literature, eight a priori determined factors were investigated: sex, residence, comorbidity, internal displacement, negative experience, family size, grade, and social indicator (SI). Comorbidity was based on RCADS-25 scores ≥ 65 (at risk) for either anxiety or depression (‘Internalizing’ was not included as it is a more general disorder). Students were deemed internally displaced if they had moved primary residence between July 2011 and July 2016. Negative experiences were categorized into five groups based on similarity: ‘violence’, including witnessing or experiencing violence and seeing dead bodies, ‘warzone’, consisting of war-related sights and sounds such as destruction, shelling, etc., ‘displacement’, incorporating home destruction and forced eviction, ‘death’, including family or community members, and ‘other’, comprising of viewing violent images on television or social media, or seeing people begging. Grade was used rather than age due to uneven numbers at the top and bottom of the distribution. SI equalled the sum of parental occupation and educational attainment presented as quartiles. Low values (e.g. Q_1_) indicated higher social position. Work scores ranged from 1 to 10 which represented International Standard Classification of Occupations, categorizations [20]. Education was scored similarly with ‘completing university’ the lowest and ‘no schooling’ the highest. Financial assets were not recorded as the war has severely skewed income distribution and young children were unlikely to know their parents’ net worth. Family size was based on the number of siblings and parents living with the child and classified into three groups; average Syrian family (5–6 members) and those above and below this category.

Covariates identified in the univariable analysis as being significantly associated with CRIES-8 scores above threshold were used to build a multivariable logistic regression model. In this model, the presence or not of PTSD symptoms remained the dependent variable with residence used as an exposure and the other variables included as potential confounders. Odds ratios (OR) and 95% confidence intervals (CI) were calculated for both uni- and multivariable statistics and the significance level was set at *p* < 0.05 for all analysis.

Missing data for CRIES-8 were replaced by zero if less than two items were absent from any subscale [14]. Eleven participants (2.2%) had over two items missing and this CRIES data was not used. If there were less than two missing items on any RCADS25-Arabic subscale, scores were prorated by multiplying the mean of completed items by the number of items in the scale as per scoring instructions [17]. Eighteen participants (3.7%) did not provide enough data and their RCADS scores were excluded. Where possible, however, demographic data were still used despite no mental health screening scores.

## Results

**Fig. 1:**
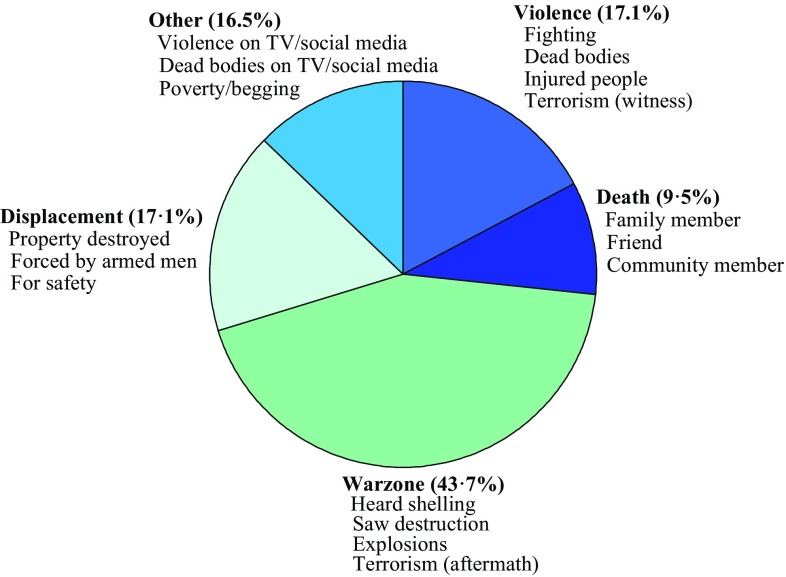
Distribution of negative experiences

A total of 492 students were recruited, 241 males and 251 females (mean age 11.4, SD 2.0, range 8–15 years). There were 247 participants from Damascus and 245 from Latakia, with five more females than males at both sites. Younger students (grades 3–5) comprised the largest age category (38%). Those reporting negative experiences totalled 158 pupils (32.1%) (Fig. 1) and Chi-square revealed that students from Damascus were more likely to do so [*χ*^2^(1, *n* = 120) = 61.71, *p* < 0.001].

Among negative experiences reported, warzone (43.7%), violence and displacement (both 17.1%) were the largest categories. However, the overwhelming majority of participants (67.9%) did not respond to this question. IDPs amounted to 50.2% of our sample (247) and were also more commonly females from Damascus [*χ*^2^(1, *n* = 142) = 7.81, *p* < 0.005 and *χ*^2^(1, *n* = 159) = 39.83, *p* < 0.001, respectively]. The majority of households (55.1%) were in the lower half of our social scale with a significant proportion of those residing in Damascus [*χ*^2^(1, *n* = 93) = 17.15, *p* < 0.001].

Females scored higher on all screening instruments and subscales than boys. Independent samples *t* tests showed that the only subscale with no statistical sex difference was for avoidance on the CRIES-8 (Table 1). High scores translated into more probable PTSD and depression cases amongst girls although percentage wise, fewer females had probable anxiety than males (28.9 vs. 30.0%) (Table 2).

**Table 1:** Mental health screening outcomes and scores for Syrian 8‒15 year-olds

	Male (*n* = 236)	Female (*n* = 245)	Total (*n* = 481)
CRIES-8 (≥ 17)	70 (29.7% [23.8‒35.5])	99 (40.4% [34.3‒46.6])	169 (35.1% [30.9‒39.4])
Intrusion^†^	6.1 (4.3)	7.8 (4.7)	7.0 (4.6)
Avoidance	7.0 (4.7)	7.6 (5.0)	7.3 (4.9)
Intrusion + avoidance^α^	13.1 (7.8)	15.4 (8.2)	14.3 (8.1)

	Male (*n* = 234)	Female (*n* = 240)	Total (*n* = 474)
RCADS-25 (≥ 65)^a^	97 (41.6%) [35.3‒48.0]	108 (44.8%) [38.4‒50.9]	205 (43.2%) [38.7‒47.6]
Depression^†^	10.7 (5.9)	12.9 (5.6)	11.8 (5.8)
Anxiety^†^	16.3 (7.9)	20.0 (7.8)	18.2 (8.1)
Internalizing^†^	27.0 (13.8)	32.9 (12.6)	30.0 (13.0)

Data are: frequency (% of cohort) [confidence interval] or mean raw score (SD)

^α^Significant sex difference *p* < 0.002

^†^Significant sex difference *p* < 0.0001

^a^For either depression or anxiety

**Table 2:** Demographic distribution of participants

	Sex		Residence	
	Male (*n* = 241)	Female (*n* = 251)	Damascus (*n* = 247)	Latakia (*n* = 245)
Age group				
1	94 [32.9‒45.2]	93 [31.1‒43.0]	89 [30.0‒42.0]	98 [33.9‒46.1]
2	80 [27.3‒39.1]	74 [23.8‒35.1]	78 [25.8‒37.4]	76 [25.2‒36.8]
3	66 [21.8‒33.0]	85 [28.0‒39.7]	80 [26.6‒38.2]	71 [23.3‒34.7]
Experience				
Yes	64 [21.0‒32.1]	94 [31.5‒43.4]	120^β^ [42.4‒54.8]	38 [11.0‒20.0]
No	176 [67.4‒78.6]	158 [57.0‒68.9]	127 [45.2‒57.7]	207 [80.0‒89.0]
IDP				
Yes	105 [37.3‒49.8]	142^β^ [50.4‒62.7]	159^β^ [58.4‒70.3]	88 [29.9‒41.9]
No	142 [52.7‒65.1]	110 [37.7‒50.0]	88 [29.7‒41.6]	157 [58.1‒70.1]
SI				
Q_1_	61 [20.2‒31.2]	50 [15.0‒24.9]	47 [14.1‒23.9]	64 [20.6‒31.6]
Q_2_	56 [17.9‒28.6]	60 [18.6‒29.2]	47 [14.1‒23.9]	69 [22.5‒33.8]
Q_3_	56 [17.9‒28.6]	62 [19.4‒30.0]	60 [18.9‒29.6]	58 [18.4‒29.0]
Q_4_	67 [22.1‒33.5]	80 [26.1‒37.6]	93^†^ [31.6‒43.7]	54 [16.9‒27.2]
Family size				
1	87 [30.0‒42.2]	77 [25.0‒36.4]	81 [26.9‒38.6]	83 [28.0‒39.8]
2	89 [30.8‒43.0]	106 [36.1‒48.3]	99 [34.0‒46.2]	96 [33.1‒45.3]
3	64 [21.0‒32.1]	69 [22.0‒33.0]	67 [21.6‒32.7]	66 [21.4‒32.5]
Comorbidity				
Depression	66 [22.5‒34.1]	86 [29.5‒41.6]	75 [24.6‒36.1]	77 [25.6‒37.2]
Anxiety	70 [24.2‒35.9]	70 [23.2‒34.6]	65 [20.8‒31.8]	75 [24.8‒36.4]

Data are: frequency [confidence interval]

Family size: 1 = below average, 2 = average, 3 = above average. Age group: 1 = 8‒10 years, 2 = 11‒12 years, 3 = 13‒15 years

*IDP* internally displaced person, *SI* social indicator, *Q* quartile

^β^Significant following Bonferroni correction *p* < 0.0125

^†^Significant following Bonferroni correction *p* < 0.00625

**Fig. 2:**
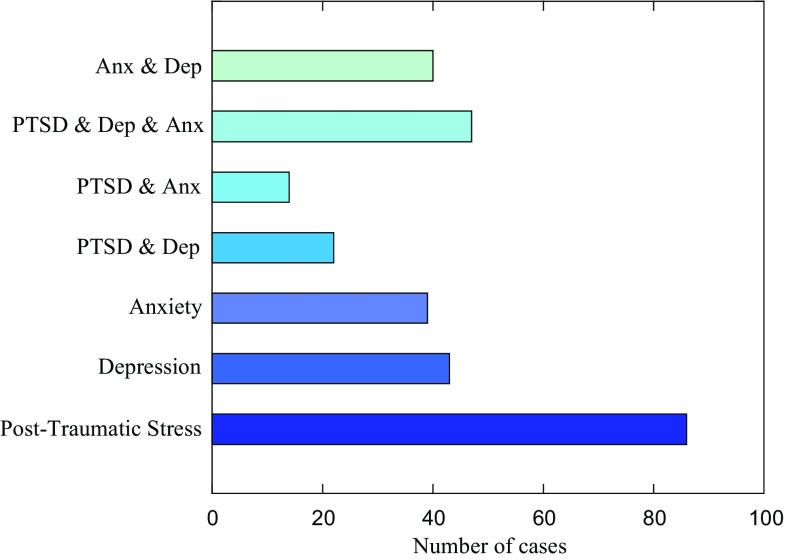
Cases of probable psychopathology including comorbidity

There were 291 participants (60.5%) above threshold for at least one probable psychological disorder with PTSD the most common (35.1%), followed by depression (32.0%) and anxiety (29.5%). Comorbid symptoms affected 153 participants (31.8%) ranging from 47 cases of ‘depression, anxiety and PTSD’ (9.8%) to 22 instances of ‘PTSD and anxiety’ (4.6%) (Fig. 2). PTSD was the most frequent unique presentation (17.9%) with anxiety the least (8.1%).

Five variables were significantly associated with PTSD symptoms following univariable analysis (Table 3). These were: sex, residence, comorbidity, internal displacement, and negative experience which were used in the multivariable logistic regression. The multivariable results showed that, following adjustment for sex, comorbidity, internal displacement, and negative experience, people living in Damascus were over two times more likely to show PTSD symptoms than those in Latakia (OR 2.36, CI 1.51–3.69, *p* < 0.001). Children showing both depression and anxiety symptoms over two and a half times more likely to have probable PTSD than those with ‘no disorder’ (the reference) (OR 2.55, CI 1.48–4.40, *p* < 0.001). Females were one and a half times more likely to indicate probable.

**Table 3:** Binary logistic regression for variables associated with post-traumatic stress symptoms

Variables	Univariable analyses			Multivariable analysis	
	PTSD (%)	OR (95% CI)	*p* value	OR (95% CI)	*p* value
Sex^a^					
Male	70 (29.7)	1		1	‒
Female	99 (40.4)	1.61 (1.10–2.35)	0.014	1.54 (1.02–2.34)	0.042
Residence					
Latakia	59 (24.2)	1		1	‒
Damascus	110 (46.4)	2.72 (1.84–4.01)	0.001	2.36 (1.51–3.69)	0.001
Comorbidities^a^					
None	80 (29.9)	1	0.001	1	0.003
One	42 (33.9)	1.20 (0.76–1.90)	0.424	1.30 (0.80–2.12)	0.288
Two	41 (52.6)	2.60 (1.56–4.36)	0.001	2.55 (1.48–4.40)	0.001
IDP^a^					
No	68 (28.3)	1		1	‒
Yes	101 (41.9)	1.83 (1.25–2.67)	0.002	1.21 (0.79–1.86)	0.389
Experience^a^					
None	98 (29.9)	1	0.001	1	0.036
Violence	6 (23.1)	0.70 (0.27–1.81)	0.466	0.52 (0.20–1.37)	0.187
Warzone	35 (53.0)	2.65 (1.55–4.54)	0.001	1.84 (1.02–3.30)	0.042
Death	7 (46.7)	2.05 (0.73–5.82)	0.176	1.76 (0.58–5.29)	0.316
Displacement	16 (61.5)	3.76 (1.65–8.57)	0.002	2.67 (1.08–6.60)	0.034
Other	7 (35.0)	1.26 (0.49–3.26)	0.629	0.77 (0.28–2.10)	0.611
Family size					
Below average	55 (34.8)	1	0.845		
Average	65 (34.0)	0.97 (0.62–1.51)	0.879		
Above average	49 (37.1)	1.11 (0.68–1.79)	0.683		
Social indictor					
Q_1_	39 (36.4)	1	0.489		
Q_2_	38 (33.0)	0.86 (0.50–1.50)	0.594		
Q_3_	36 (30.8)	0.76 (0.44–1.35)	0.369		
Q_4_	56 (39.4)	1.14 (0.68–1.91)	0.631		
Grade					
Third	37 (45.7)	1	0.367		
Fourth	24 (34.3)	0.62 (0.32–1.20)	0.156		
Fifth	22 (28.9)	0.48 (0.25–0.94)	0.032		
Six	27 (33.8)	0.61 (0.32–1.15)	0.123		
Seventh	17 (28.8)	0.48 (0.24–0.98)	0.044		
Eight	20 (38.5)	0.74 (0.37–1.51)	0.412		
Ninth	22 (34.9)	0.64 (0.32–1.26)	0.194		

Model summary for multivariable analysis: − 2 Log likelihood = 548.35

Cox and Snell *R* square = 0.12, Nagelkerke *R* square = 0.16. Hosmer and Lemeshow, Chi-square value = 8.18, *p* = 0.42

*OR* = odds ratio, *CI* = confidence interval, *IDP* = internally displaced persons, *Q* = quartile

^a^Potential confounders

PTSD (OR 1.54, CI 1.02–2.34, *p* < 0.04) with negative events also predict PTSD symptoms. “Displacement experience” increased the chances of probable PTSD over two and a half times and warzone experiences, close to double the reference (no experience) (OR 2.67, CI 1.08–6.60, *p* < 0.03 and OR 1.84, CI 1.02–3.30, *p* < 0.04) (Table 3). An unforeseen finding was that being internally displaced did not predict PTSD symptoms in the multivariable model (OR 1.21, CI 0.79–1.86, *p* < 0.39).

## Discussion

This study aimed to document the mental health of Syrian 8- to 15-year-old schoolchildren remaining within the country’s borders. Empirical data are presented from two parts of Syria which show the incidence of symptoms for three common mental health problems associated with war: PTSD, depression, and anxiety. In addition, the study provides evidence of protective and risk factors for PTSD symptoms.

Overall 60.2% of participants scored above threshold for one or more psychological condition which is consistent with studies from conflict areas [16, 21]. Cases of probable PTSD (35.1%) were greatly elevated compared to the general population [22]. However, comparisons with pre-crisis Syrian mental health statistics were not possible through lack of data. The PTSD incidence reported here is analogous to findings with Syrian child refugees which range from 30 to 45% [11, 12]. In contrast to regional conflict areas such as Gaza, where PTSD symptoms have been reported in as many as 70% of participants [16], the number of cases in this study can be considered low. The amount of students scoring above threshold for anxiety symptoms (29.5%) was similar to percentages reported elsewhere [23], but this was not the case for depression (32.0%) where rates were half that of young Syrian refugees [11]. This difference may relate to contrasts in day-to-day stressors between populations and the protective aspects of daily routine for those who remain such as, family networks, school, local familiarity, etc. These factors have been identified as being important in the formation of resilience to psychological distress in war-affected children. For example, Betancourt and Kahn [24] described resilience as moulded by the interaction between a child’s individual characteristics, family life and the larger social environment (school and neighbourhood as well as political and cultural considerations). It is uncomplicated, therefore, to see how these mediating elements are disrupted through separation from the home environment and helps to explain the discrepancy between our findings and those interviewed outside of the country. Students from Damascus were more likely to reach threshold for PTSD symptoms than their counterparts in Latakia. This finding was anticipated due to the proximity of fighting which is known to predict mental health problems [25]. Frontlines brought continuous warzone sights and sounds, as well as fears of shelling or terrorist attack to the capital and undoubtedly increased stress and fear responses. The military situation in Damascus at the time, also explains the significant number of IDPs in our sample from that area, as densely inhabited districts and surrounding countryside were depopulated by fighting. Nevertheless, Latakia also has a large IDP population with many new residents having fled violence in Latakia province, Idlib and Aleppo. It is unsurprising, therefore, that the rate of PTSD symptoms in this city was also found to be higher than general populations [22].

Unexpectedly, few children reported a negative experience (32.1%) despite the high levels of potential psychopathology recorded. One explanation is that students witnessed negative experiences on a daily basis (rather than individual traumatic events) and were therefore unsure how to respond to the question. Our data support this proposition as ‘warzone’ was the largest category of negative experience. The large number of non-responses may also be attributable to children being uncomfortable disclosing their experiences for personal and/or social reasons. Older children answered this question less (data not shown) suggesting this may be the case as normally, they would be better placed to articulate events.

Another unanticipated finding regarding negative experiences was the number of children who described witnessing violence through social media or television. Media violence is known to play a role in forming and maintaining psychological distress, particularly in youths [26], but given the on-going conflict around, it was not expected to feature in our results. However, in Syria, the crisis dominates news bulletins and the aftermath of terrorist atrocities, fighting, and dead or injured people are regularly shown. Social media has also been a significant tool for propagating information since the outbreak of the war. Islamist groups, in particular, have used the major internet communication platforms to distribute images and videos of executions, suicide attacks, etc. The availability and effects of these images on children warrants further study worldwide but within the context of Syria, our data suggest the proliferation of such material is widespread amongst young people and may be causing serious harm.

Some negative events predicted PTSD symptoms, but the types of experience were not typical. Death and violence are the most common predictors of mental health problems [21, 27], but neither was significant in our study. Both were potentially underreported as children could have been reluctant to communicate such events via self-report measure or in front of non-family members. Instead, warzone and displacement were most strongly associated with PTSD symptoms. The category warzone includes many ‘low-level’ conflict stressors such as hearing shelling or seeing destroyed buildings. That warzone predicted PTSD symptoms, supports those who argue that continuous exposure to conflict environments can have the same negative outcomes as major traumatic events [13, 16]. Regarding displacement, it was unusual to find ‘displacement experience’ predicted PTSD symptoms but not being an IDP. Nevertheless, people were displaced for diverse reasons with some moving voluntarily for safety and others because of properties being destroyed. Students also reported homes being seized by armed groups. It is suggested that IDPs in these latter scenarios were more likely to report displacement as a traumatic event with PTSD symptomology a potential consequence.

A noteworthy outcome of the univariable analysis relates to factors that did not predict PTSD. No sub-group within family size, social indicator, or grade/age, influenced PTSD demonstrating the universal effect the crisis has had on Syrian society. Some of these factors influence resilience mechanisms (see above) for example, large families can be protective for mental health as they increase the chances of having a positive sibling relationship [28]. In conflict situations, however, large families are more likely to have a member directly involved or affected by the conflict and providing financially becomes more challenging. Social status also guards against psychopathology [1, 29]. Nonetheless, in long-term conflicts where violence is random and resources become scarce (such as in Syria), social structure becomes less significant [7, 13]. Age has long been understood to predict psychological distress and it has been consistently reported that 13–18 year-olds are more susceptible to mood disorders than younger children [30]. However, the majority of this research has been with non-conflict populations. That no differences were found in our research supports a proposition that age is less protective for mental health when environmental stressors are greater and more prevalent [31].

Four predictors of PTSD in Syrian children were identified through the multivariable logistic regression. Following adjustment, residence remained a significant predictor of potential PTSD as did sex, comorbidity and negative experience.

Sex differences in mental health are well established [32]. In agreement with other studies, it was found that females were more likely to display PTSD and depression symptoms than males although there was little difference regarding anxiety (Table 2) [16, 26, 29]. Anxiety and, in particular, depression, are known to co-occur with PTSD and nearly a third of our sample were comorbid. Several theories for why these disorders regularly co-occur have been advanced. One is that the three psychopathologies share common risk factors [33]. Given the conflict setting of our sample this is possible; however, individual instances of each disorder were greater than comorbid cases. Another hypothesis is that PTSD leads to anxiety and depression [27]. If this was the case, anxiety and depression should have been higher in Damascus compared to Latakia as cases of PTSD symptoms were greater in the capital. However, the two disorders were evenly distributed across cities (Table 2). Be that as it may, fewer students were comorbid in Damascus and it is hypothesized that daily exposure to the conflict (and the resulting high PTSD symptomology), ‘masked’ other symptoms that may appear after time, away from hostilities [13]. Regardless of the origins of the high CRIES-8 scores in our study, the large number of comorbid cases identified suggests that Syrian children should be routinely screened for multiple disorders.

Our data clearly show that Syria has a current and looming mental health crisis that needs to be addressed. The on-going nature of the conflict, however, complicates the successful implementation of appropriate mental health interventions. By conducting research within the school system, we have highlighted the merits of using brief screening instruments in this setting to ascertain incidence and risk factors associated with child psychopathology [34]. Working in schools also demonstrates their potential for being used to implement preventative and/or rehabilitation programmes. The efficacy of school-based interventions in reducing psychological distress has been established in different cultures and conflicts [35] and Syrian schools have previously been identified as a place to provide mental health services to refugees. Following the US/UK invasion in 2003, large numbers of Iraqis entered Syria and many presented with mental health issues. In response, international agencies and relevant Syrian institutions initiated a framework to implement MHPSS countrywide emphasizing schools as a key aspect of delivery [36]. An advantage of using Syrian state schools is that they are mixed vis-à-vis social class and religion, meaning mental health providers can deliver services to all aspects of the community. Whilst the infrastructure to provide services through the school system is potentially in place, there is a shortage of qualified staff to initiate such programmes in Syria, a situation that must be challenged if Syria’s future generations are to be supported.

The results of this study better inform MHPSS professionals about the scale of the crisis inside Syria and provide an indication of the likely mental health outcomes for children and Syrian society in the future. The findings are important for those coordinating the MHPSS response within the country and for independent international aid organizations that wish to support ordinary Syrians now or once hostilities have ended.

## Study limitations

There are a number of limitations to this study. Our school selection process was governed by what was feasible given the security situation in large areas of the country. Our data also fail to include children who could not attend school through lack of access, had been withdrawn by their parents or did not wish to participate. There are several additional, well-studied risk and protective factors for mental well-being. Correlations between parental and child mental health, as well as family functioning, are important examples [24]. The study, therefore, would have benefited from further data collection with parents/caregivers. In addition, the individual factors that influence resilience are of great interest to child mental health researchers. A more in-depth understanding of participant’s personality, beliefs and characteristics would, therefore, have been preferable. However, due to time, personnel, practical, and financial constraints, further data collection was beyond the scope of this project.

## References

[1] Saxena S, Thornicroft G, Knapp M, Whiteford H (2007). Resources for mental health: scarcity, inequity, and inefficiency. Lancet.

[2] United Nations Office for the Coordination of Humanitarian Affairs (UNOCHA) (2017) Syria crisis overview. http://www.unocha.org/syrian-arab-republic/syria-country-profile/about-crisis. Accessed 2 Aug 2017

[3] United Nations Security Council statement S/PRST/2015/15 (2015) Alarmed by continuing Syria crisis—security council affirms its support for special envoy’s approach in moving political solution forward. https://www.un.org/press/en/2015/sc12008.doc.htm. Accessed 28 July 2017

[4] United Nations Office for the Coordination of Humanitarian Affairs (UNOCHA) (2016) Humanitarian needs overview—Syrian Arab Republic. https://reliefweb.int/sites/reliefweb.int/files/resources/2016_hno_syrian_arab_republic.pdf. Accessed 21 July 2017

[5] Sharara SL, Kanj SS (2014). War and infectious diseases: challenges of the Syrian civil war. PLoS Pathog.

[6] Walker J (2016) Study on humanitarian impact of Syria—related unilateral restrictive measures. https://assets.documentcloud.org/documents/3115191/Hum-Impact-of-Syria-Related-Res-Eco-Measures-26.pdf. Accessed 18 Oct 2017

[7] Ben Taleb Z, Bahelah R, Fouad FM, Coutts A, Wilcox M, Maziak W (2015). Syria: health in a country undergoing tragic transition. Int J Public Health.

[8] Tol WA, Barbui C, Galappatti A, Silove D, Betancourt TS, Souza R (2011). Mental health and psychosocial support in humanitarian settings: linking practice and research. Lancet.

[9] Coutts A, Fouad FM, Abbara A, Sibai AM, Sahloul Z, Blanchet K (2015). Responding to the Syrian health crisis: the need for data and research. Lancet Respir Med.

[10] Bashour H (2015). Let’s not forget the health of the Syrians within their own country. Am J Public Health.

[11] Özer S, Şirin S, Oppedal B (2013) Bahçeşehir study of Syrian refugee children in Turkey. https://www.fhi.no/globalassets/migrering/dokumenter/pdf/bahcesehir-study-report3.pdf. Accessed 11 June 2017

[12] Soykoek S, Mall V, Nehring I, Henningsen P, Aberl S (2017). Post-traumatic stress disorder in Syrian children of a German refugee camp. Lancet.

[13] Miller KE, Rasmussen A (2014). War experiences, daily stressors and mental health five years on: elaborations and future directions. Intervention.

[14] Perrin S, Meiser-Stedman R, Smith P (2005). The Children’s Revised Impact of Event Scale (CRIES): validity as a screening instrument for PTSD. Behav Cogn Psychother.

[15] American Psychiatric Association (1994). Diagnostic and statistical manual of mental disorders.

[16] Thabet AA, Abu Tawahina A, El Sarraj E, Vostanis P (2008). Exposure to war trauma and PTSD among parents and children in the Gaza strip. Eur Child Adolesc Psychiatry.

[17] Chorpita B, Ebesutani C (2014) Revised Children’s Anxiety and Depression Scale users guide. http://www.childfirst.ucla.edu/RCADSUsersGuide20140711.pdf. Accessed 28 Apr 2017

[18] Stevanovic D, Bagheri Z, Atilola O, Vostanis P, Stupar D, Moreira P (2017). Cross-cultural measurement invariance of the Revised Child Anxiety and Depression Scale across eleven world-wide societies. Epidemiol Psychiatr Sci.

[19] IBM Corp. Released. IBM SPSS Statistics for Windows, Version 22.0. 2013. IBM Corp, Armonk

[20] International Labour Office (2007) International Standard Classification of Occupations ISCO-08. Resolution concerning updating the International Standard Classification of Occupations. http://www.ilo.org/public/english/bureau/stat/isco/docs/resol08.pdf. Accessed 15 June 2017

[21] Claycomb MA, Charak R, Kaplow J, Layne CM, Pynoos R, Elhai JD (2016). Persistent complex bereavement disorder symptom domains relate differentially to PTSD and depression: a study of war-exposed Bosnian adolescents. J Abnorm Child Psychol.

[22] Polanczyk GV, Salum GA, Sugaya LS, Caye A, Rohde LA (2015). Annual Research Review: A meta-analysis of the worldwide prevalence of mental disorders in children and adolescents. J Child Psychol Psychiatry.

[23] Ugurlu N, Akca L, Acarturk C (2016). An art therapy intervention for symptoms of post-traumatic stress, depression and anxiety among Syrian refugee children. Vulnerable Child Youth Stud.

[24] Betancourt TS, Kahn KT (2008). The mental health of children affected by armed conflict: protective processes and pathways to resilience. Int Rev Psychiatry.

[25] Thabet AAM, Vostanis P (1999). Post-traumatic stress reactions in children of war. J Child Psychol Psychiatry.

[26] Otto MW, Henin A, Hirshfeld-Becker DR, Pollack MH, Biederman J, Rosenbaum JF (2007). Posttraumatic stress disorder symptoms following media exposure to tragic events: impact of 9/11 on children at risk for anxiety disorders. J Anxiety Disord.

[27] Breslau N (2012). Epidemiology of posttraumatic stress disorder in adults. The Oxford handbook of traumatic stress disorders.

[28] Grinde B, Tambs K (2016). Effect of household size on mental problems in children: results from the Norwegian mother and child cohort study. BMC Psychol.

[29] Betancourt TS, Borisova II, de la Soudière M, Williamson J (2011). Sierra Leone’s child soldiers: war exposures and mental health problems by gender. J Adolesc Health.

[30] Costello EJ, Erkanli A, Angold A (2006). Is there an epidemic of child or adolescent depression?. J Child Psychol Psychiatry.

[31] Lonigan CJ, Shannon MP, Taylor CM, Finch A, Sallee FR (1994). Children exposed to disaster: II. Risk factors for the development of post-traumatic symptomatology. J Am Acad Child Adolesc Psychiatry.

[32] Rosenfield S, Mouzon D, Aneshensel CS, Phelan JC, Bierman A (2013). Gender and mental health. . Handbooks of sociology and social research. Handbook of the sociology of mental health.

[33] Spinhoven P, Penninx BW, van Hemert AM, de Rooij M, Elzinga BM (2014). Comorbidity of PTSD in anxiety and depressive disorders: prevalence and shared risk factors. Child Abuse Negl.

[34] Panter-Brick C, Eggerman M, Gonzalez V, Safdar S (2009). Violence, suffering, and mental health in Afghanistan: a school-based survey. Lancet.

[35] Barry MM, Clarke AM, Jenkins R, Patel V (2013). A systematic review of the effectiveness of mental health promotion interventions for young people in low and middle-income countries. BMC Public Health.

[36] Quosh C (2011). Takamol: multi-professional capacity building in order to strengthen the psychosocial and mental health sector in response to refugee crises in Syria. Intervention.

